# Effective process or dangerous precipice: qualitative comparative embedded case study with young people with epilepsy and their parents during transition from children’s to adult services

**DOI:** 10.1186/1471-2431-13-169

**Published:** 2013-10-16

**Authors:** Sheila A Lewis, Jane Noyes

**Affiliations:** 1 Centre for Health-Related Research, School of Healthcare Sciences, Bangor University, Bangor LL57 2EF, UK

**Keywords:** Young people, Parents, Epilepsy, Transition, Qualitative case-study, Theory-based evaluation, Communication, Information needs, Knowledge exchange, Epilepsy nurse specialist

## Abstract

**Background:**

Transition from children’s to adult epilepsy services is known to be challenging. Some young people partially or completely disengage from contact with services, thereby risking their health and wellbeing. We conducted a mixed-method systematic review that showed current epilepsy transition models enabling information exchange and developing self-care skills were not working well. We used synthesised evidence to develop a theoretical framework to inform this qualitative study. The aim was to address a critical research gap by exploring communication, information needs, and experiences of knowledge exchange in clinical settings by young people and their parents, during transition from children’s to adult epilepsy services.

**Method:**

Qualitative comparative embedded Case study with 2 'transition’ cases (epilepsy services) in two hospitals. Fifty-eight participants: 30 young people (13–19 years) and 28 parents were interviewed in-depth (individual or focus group). Clinical documents/guidelines were collated. 'Framework’ thematic analysis was used. The theoretical framework was tested using themes, pattern matching and replication logic. Theory-based evaluation methods were used to understand how and why different models of service delivery worked.

**Results:**

A joint epilepsy clinic for young people 14–17 years coordinated by children’s and adult services was more likely to influence young people’s behaviour by facilitating more positive engagement with adult healthcare professionals and retention of epilepsy-related self-care information. Critical success factors were continuity of care, on-going and consistent age-appropriate and person centred communication and repeated information exchange. Three young people who experienced a single handover clinic disengaged from services. Psychosocial care was generally inadequate and healthcare professionals lacked awareness of memory impairment. Parents lacked knowledge, skills and support to enable their child to independently self-care. Translation of transition policies/guidelines into practice was weak.

**Conclusion:**

Findings make a significant contribution to understanding why young people disengage from epilepsy services, why some parents prevent independent self-care, and what constitutes good communication and transition from the perspective of young people and parents. The type of service configuration, delivery and organisation influenced the behaviours of young people at transition to adult services. The novel theoretical framework was substantially supported, underwent further post-hoc development and can be used in future practice/intervention development and research.

## Background

Epilepsy is a common long-term neurological condition affecting approximately 5–7 cases per 10,000 children and young people every year [1]. Epilepsy has life-long consequences and children require a process of transition to adult epilepsy services for their on-going life-course management. Young people require high levels of self-care and self-management skills to achieve effective epilepsy control, and optimal epilepsy-related quality of life and general wellbeing. The adolescent years are associated with many specific challenges, including striving for independence. There are global reports [2,3], backed up by our own clinical experience, of young people partially or fully disengaging with health services to 'go it alone’ without support from healthcare professionals during the transition from children’s to adult services. Any degree of disengagement from epilepsy services could have serious consequences for the health and wellbeing of young people and presents a major challenge to health service providers.

Transition models and pathways between children’s and adult epilepsy services have been designed for use in many developed country contexts [4,5]. Children’s services in the United Kingdom (UK) are mostly designed for children under 19 years and there is some international variation in the age at which adult services commence. Little is known about this critical period of time in the lives of young people or why some young people disengage partially or fully with treatment and professional support. Addressing this gap in knowledge is critical to the development of effective epilepsy services that enable young people to stay engaged with professionals, optimally self-care and manage their epilepsy during transition and beyond. The purpose of the study reported here is to start addressing this significant gap in knowledge.

### Theoretical Framework

We previously undertook a mixed-method systematic review [6] to develop the theoretical framework and propositions used to inform the qualitative Case study reported here. The theoretical framework represents several explanatory pathways and mechanisms as to how young people engage in communication and information exchange with healthcare professionals in routine clinical encounters. At its most basic, we hypothesised that young people with epilepsy who actively participate in consultations with healthcare professionals, and who are able to ask questions and receive accurate information, develop skills to manage their epilepsy and are given the freedom by their parents to lead an independent life. Whereas young people with epilepsy that do not interact and ask questions during their consultations develop misconceptions, are afraid to ask questions due to fear of negative consequences, and this may lead to dependence on their parents and potentially disengaging from services.

### Communication, information and knowledge exchange in clinical encounters

'Knowledge exchange’ in the context of this study is defined as an essential communication process of exchanging epilepsy information and knowledge in its broadest context between healthcare professionals, young people and their families. This communication and knowledge exchange process is conceived as vital for young people and their families to develop self-management and self-care skills. Within these specific healthcare contexts, communication between specialist epilepsy healthcare professionals and young people is conceptualised from varying conceptual, theoretical, policy and practice perspectives in literature and clinical guidance. Whilst there is conceptual clarity on intent in transition policy and clinical guidelines, there is little age-appropriate practical guidance on how to translate essential elements and aspirations (such as utilizing active listening skills with teenagers) into routine clinical practice. Observational and qualitative studies looking at child-centeredness and age-appropriate communication in clinical encounters have reported important deficiencies across all age groups [7].

### Health literacy

The current qualitative Case study was conducted alongside an extensive 7 year mixed-method programme of work looking at children’s health literacy, analysis of available condition-specific health information, and development and evaluation of a suite of educational interventions to promote long-term self-management of diabetes type 1 [8-10]. Within a wider long-term condition context, our parallel qualitative studies into health literacy suggested that condition-specific knowledge may not by itself support adherence either in childhood or as a young person. More importantly, failure to involve children and young people with long-term conditions in routine clinical encounters with healthcare professionals and decision-making appeared more likely to result in sub-optimal self-management in childhood and potential disengagement from professional support.

The ability to use condition-specific information to support decision-making and self-care is undoubtedly linked to health literacy. In this context health literacy means the degree to which children and young people have both the motivation and capacity to obtain, process and understand basic epilepsy-specific self-care and management information, and make optimal use of epilepsy services and epilepsy support to make appropriate decisions about their medicines management and lifestyle adjustments. Medication literacy encompasses the skills needed to access, understand and act on medicines information. In the parallel Information Matters Project [10] our interest in medication literacy (including epilepsy medication) was the availability, effective communication and facilitation of information to support the decisions of children, young people and their families made at home, and in their everyday lives. In contrast, the current qualitative Case study went a step further by exploring young people and their parents’ experiences of communication, information and knowledge exchange in routine clinical encounters in two epilepsy services. As our mixed-method systematic review had flagged memory impairment as an epilepsy-specific issue, we set out to specifically look for the impact of memory impairment as described by children, young people and their parents.

### Self-care and self-management

Although both terms are often used interchangeably in the literature, Kirk et al. [11] conceptualise important differences between self-care and self-management. Self-care refers to the broad range of activities people do to manage living with a long-term condition, whereas self-management relates to aspects such as condition monitoring, symptom management and the instigation of therapies and medications. Adoption and maintenance of specific behaviours is required to safely self-care and manage epilepsy [12].

### Transition from children’s to adult services

For the purposes of this qualitative Case study, we used the definition of 'transition’ that is used in UK and European policy and practice. 'Transition’ is conceptualised as the 'phase or period of time between the teens and twenties which is broken up educationally and administratively. During the transition phase there are changes of responsibility from child to adult services, from school to further and higher education and from childhood dependence to adult responsibility’[13]. In the United States (US), however we acknowledge that 'transition’ is defined and conceptualised slightly differently with variation in linguistic meaning, with 'transition’ being the process by which young people become more involved in their care and condition-specific management, whilst 'transfer’ refers to the physical movement from children’s to adult services.

The aim of this qualitative comparative embedded Case study was to explore the views of young people with epilepsy (and their parents) about their experience of communication, information and knowledge exchange in two epilepsy services. The research questions were as follows. In each Case:

● How has the service attended to the epilepsy, biological, psychosocial and educational information needs of young people with epilepsy?

● How did young people with epilepsy experience communication and information exchange with healthcare professionals in the epilepsy clinic?

● How much support and information have parents received from the service to enable them to encourage safe independent epilepsy self-care and management?

## Methods

### Design and setting

**Figure 1:**
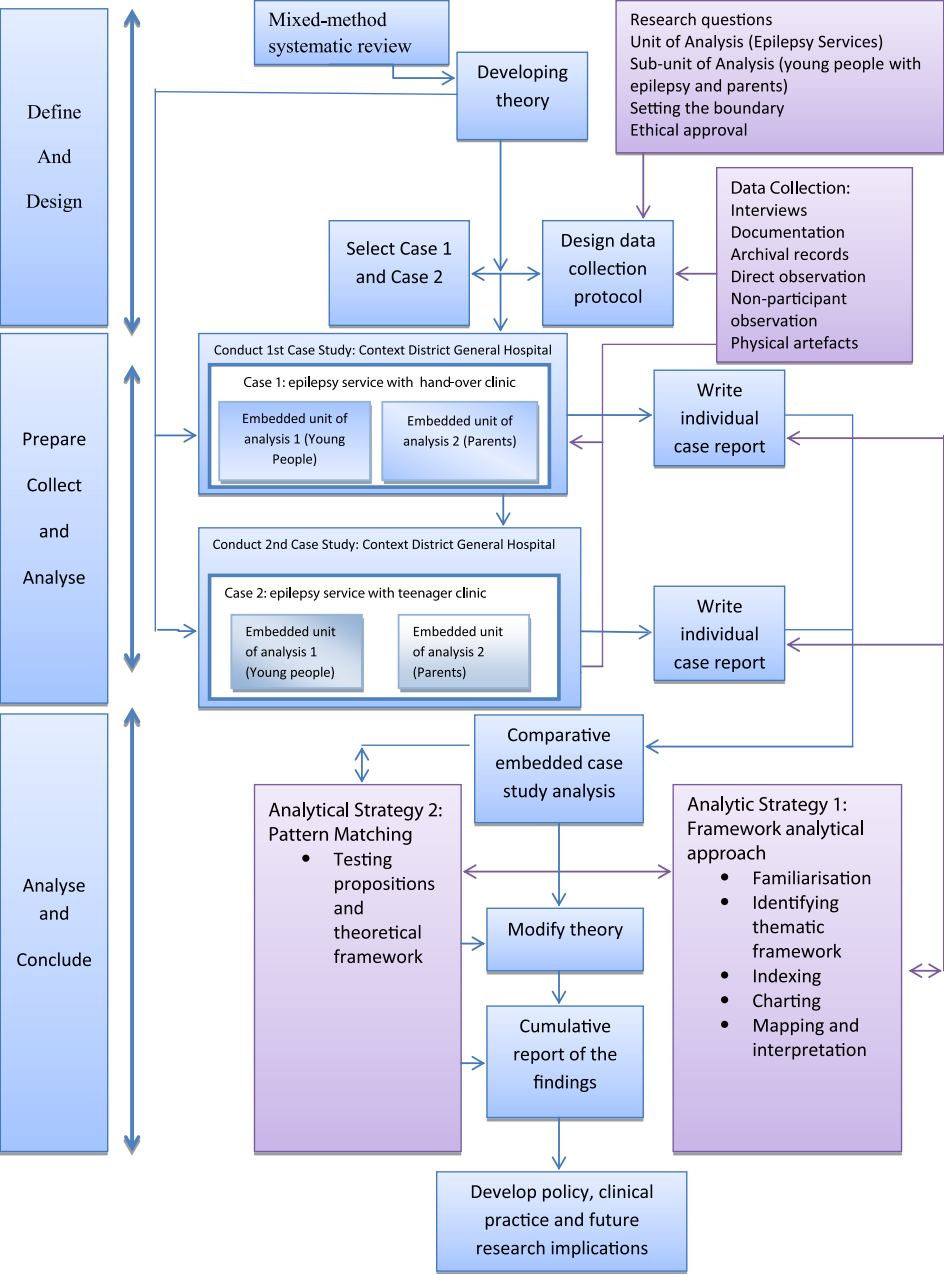
Comparative embedded qualitative Case study design.

A qualitative comparative embedded Case study (see Figure 1) [14] enables investigation of a contemporary phenomenon in depth within real-life contexts, especially where the boundaries between phenomenon and context are not clearly evident [14]. A “Case connotes a spatially delimited phenomenon observed at a single point in time or over a period of time” and can “be created out of any phenomenon so long as it has identifiable boundaries and comprises the primary object of inference” [15]. A case can be an individual, a role, a small group, an organisation or a nation [16], it can also be an event, a concept, a programme or a process [17].

The real life phenomena being explored were the epilepsy services (the case), and young people with epilepsy and their parents. The focus was specifically on communication with healthcare professionals and experience of information and knowledge exchange in different clinic settings during transition from children’s to adult services. A qualitative Case study becomes embedded when it contains more than one sub-unit of analysis. When individuals are the unit of analysis (or in this study embedded-units) their experiences are an important factor [18] and can develop rich descriptions and understanding about their experience of information and knowledge exchange in the epilepsy services. Findings are therefore more likely to have a direct influence on policy, practice and future research [19].

**Figure 2:**
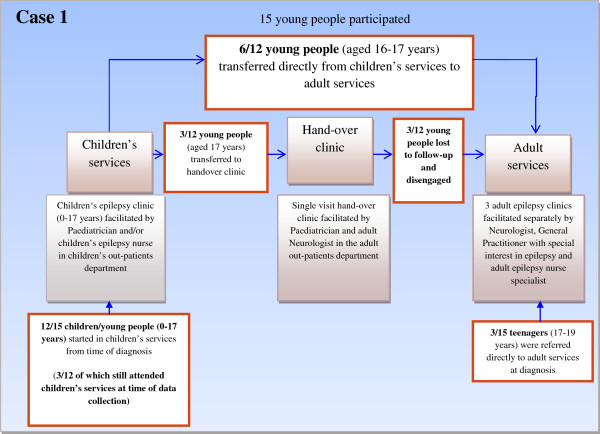
Model of transition Case 1.

**Figure 3:**
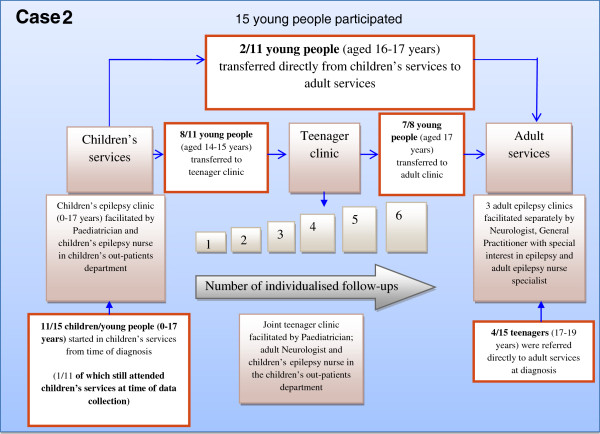
Model of transition Case 2.

The qualitative unit of analysis was therefore children’s and adult epilepsy services in two District General Hospitals (Case 1 and Case 2) and two embedded-units of analysis were young people and their parents within each Case. The different models are shown in Figures 2 and 3.

### Case 1 (please see Figure 2)

The epilepsy service programme theory in Case 1 followed a model of care delivered by children’s services until the young person reached aged 17 years. This model of care was child and family centred and inclusive of parents. When the young person reached 17 years, and it was determined that the young person needed on-going care in adult services, the programme logic was to 'handover’ the young person to adult services via a single handover clinic for epilepsy-specific care. The epilepsy service programme theory in adult services entailed the young person being seen as an adult, and able to take responsibility for their epilepsy, independent of parents. Other young people were directly transferred from the children’s clinic to the adult epilepsy clinic without experiencing a single 'handover’ clinic, and some young people who were diagnosed at 16–17 years were referred directly to the adult clinic. No rationale was available to explain the various care pathways young people experienced.

### Case 2 (please see Figure 3)

The epilepsy service programme theory in Case 2 was that young people were identified in children’s services at an early age as being in need of future care in adult services when older. The programme theory was young person centred, inclusive of parents, and between 14–17 years involved joint care in a teenager clinic by a paediatrician, adult neurologist and a children’s epilepsy nurse providing specialised epilepsy management. The children’s services in Case 2 also followed a model of care whose programme theory attended to the young person’s medical (including epilepsy), psychosocial and educational (school) needs. Care was child and family centred and inclusive of parents. The programme logic involved children’s services seeing the young person over months and years, at alternate intervals to the joint teenager clinic, to facilitate a smooth transition and provide opportunities for the young person and family to discuss any concerns.

If the young person reached 16–17 years of age and did not require on-going care in children’s services the programme logic was to transfer the young person to adult services for epilepsy-specific care. The programme theory in adult services involved the young person being viewed as an adult, and therefore able to take responsibility for their epilepsy, independent of parents. As in Case 1, some young people were transferred directly from children to adult epilepsy services and others were referred directly to the adult epilepsy clinic with no explanation about why this occurred.

### Participants

All young people aged between 13–19 years with epilepsy attending the children’s, transitional teenager, handover and adult epilepsy clinics were identified in each Case. Young people were included if they had a diagnosis of epilepsy and who had reported experience of a seizure in the past 12 months. Young people who were recruited needed to be able to participate in a young person centred interview or focus group and be able to communicate their own views and experiences from their own perspective. Young people with severe learning disabilities (and their parents) were not included in this study as they were considered a different group with specific information and communication needs. Young people with severe learning disabilities are generally reliant on their parents for engagement and participation in epilepsy services with healthcare professionals, and usually do not have the capacity to disengage from services by themselves. Parents of young people with epilepsy who met the inclusion criteria were also invited to participate. Written informed consent was obtained from young people over 16 years and parents. Written informed assent was obtained from young people under 16 years and, in addition, written informed proxy consent was obtained from parents/guardians.

### Data collection

Qualitative Case study research methodology is characterized by multiple data collection methods through triangulation, which strengthens the validity of the findings [14]. Gathering rich, contextual data from multiple sources about the phenomenon provides a holistic and deeper understanding of real-life contexts and is considered to be more rigorous when compared to other qualitative methods [20]. Primary sources were individual, small group interviews and focus groups [21]. Young people and parents chose the mode of interview most suitable to themselves. Secondary sources included documentation, archival records and physical artefacts. Following the process of replication logic, data were first collected in Case 1 and then replicated in Case 2 in exactly the same way.

### Data analysis

To achieve a high quality qualitative analysis four main principles were maintained:

● All evidence collected from multiple sources was included;

● All rival explanations were considered;

● The most important findings were presented in this context; and

● The researchers’ personal experiences and expert epilepsy knowledge was used to interpret the evidence [14].

**Figure 4:**
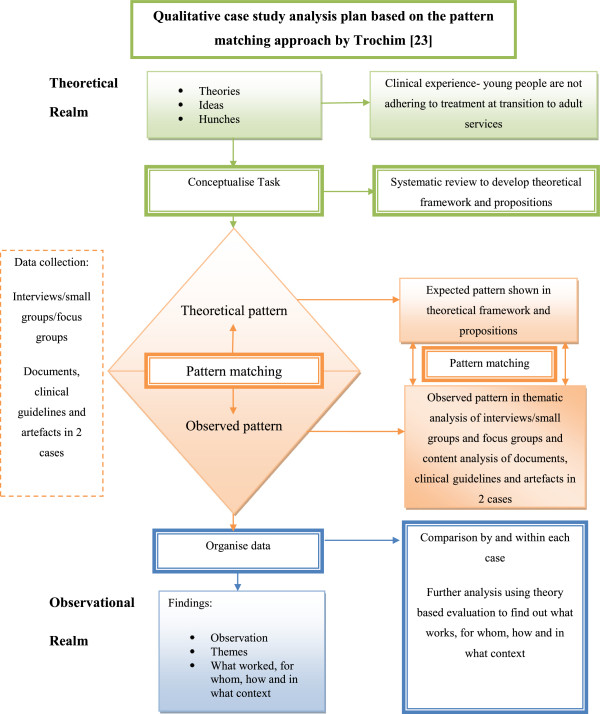
**Pattern Matching (Trochim, 1989 **[23]**).**

A two-stage analytical approach was guided by the theoretical framework and propositions [6]. First, thematic analysis using the Framework approach [22] was used to explore experiences of young people with epilepsy and their parents attending epilepsy services in Case 1 and Case 2. Second, pattern matching [23] (Figure 4) using theory-based evaluation was applied to test the theoretical framework and propositions and better understand which particular models of service delivery worked during transition to adult services, for whom, and in what contexts.

The 'Framework’ is an analytical approach for applied policy research with five stages:

● Familiarisation;

● Identifying a thematic framework;

● Indexing;

● Charting; and

● Mapping and interpretation

**Figure 5:**
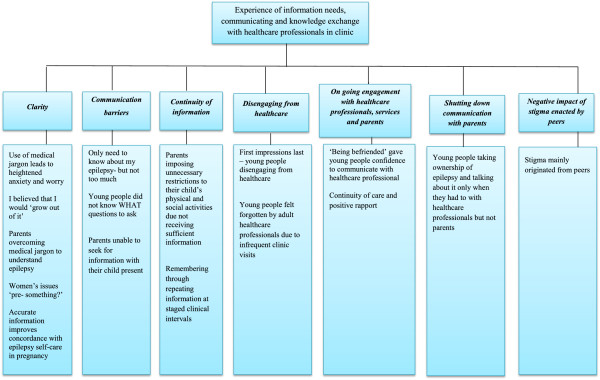
Themes from comparative embedded qualitative Case study.

Relevant content of retrieved documents and archival records were analysed using qualitative content analysis [24]. The presence and/or absence of documents helped identify which models of service delivery, guidelines, policies and processes were being used in healthcare contexts in the two transition cases. Interviews and focus groups were transcribed verbatim. Other narrative data retrieved from documents and archival records were collected and sorted. Computer software [25] was used to manage narrative data. Thematic analysis was conducted within each case, and then across the two transition cases. Following familiarisation with the evidence, an a priori index of codes (Figure 5- thematic coding diagram) was developed, informed by the study theoretical framework and propositions [6]. Index codes were then applied to the evidence. Interpretation of evidence was guided by creating charts and maps for further interrogation, discussion and refinement.

Pattern matching [23] (Figure 4) involved matching expected patterns in the theoretical framework and propositions to observed patterns in the qualitative comparative embedded case study themes. We used a deductive approach by applying each Case study theme to the theoretical framework and each theoretical proposition, noting patterns and any clustering of ideas to determine whether case study themes agree or disagree (rival explanation) with the propositions. Theoretical 'expected’ patterns were considered to be a 'series of benchmarks’ against which observed patterns were compared. Yin [14] talks about 'significant’ findings, but in this qualitative context he actually refers to presenting the most 'important’ findings.

We then used Miles and Huberman’s [16] tactics for generating meaning and theoretical coherence by adopting theory-based evaluation methods to interrogate findings from pattern matching. This approach enabled better understanding of which particular models of service delivery worked during transition to adult services, for whom, and in what contexts. We adopted both Pawson and Tilley [26], and similar positions on mechanisms of action as summarised by Asbury and Leeuw [27] that 'Interventions such as epilepsy service models work at transition (have successful outcomes) only in so far as they introduce appropriate ideas and opportunities (mechanisms) for people (children, parents, professionals) in the appropriate social and cultural conditions (contexts). The mechanism of change is not the intervention (*epilepsy service model and components*), but the behavioural response that the intervention and associated activities generate’.

### Post-hoc theory development

**Figure 6:**
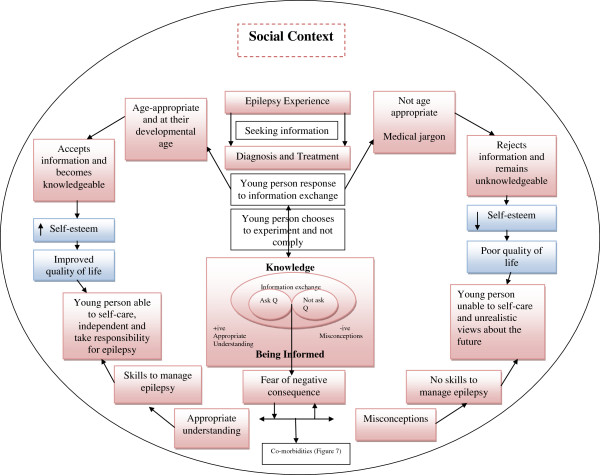
Theoretical Framework post-hoc development.

**Figure 7:**
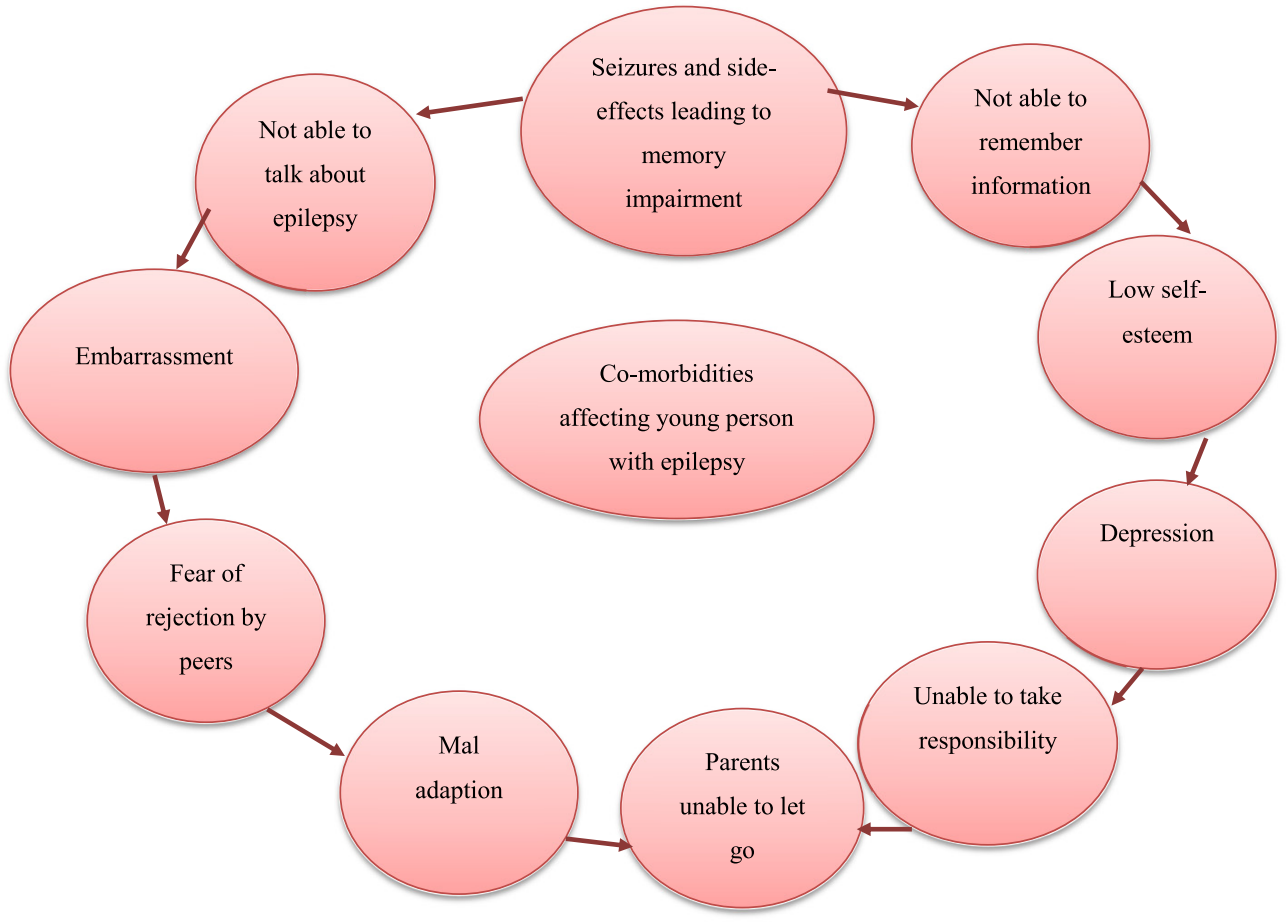
Co-morbidities experienced by young people as a result of having epilepsy.

At the end of the analysis we had an overall picture of where expected patterns in the theoretical framework (Figures 6 and 7) and propositions (Table 1) matched with observed patterns in Case study data. These data were used to support and further develop the theoretical framework and a programme theory to inform future intervention and practice development.

**Table 1:** Propositions post-hoc development

1	Age appropriate psychosocial-educational programmes for young people with epilepsy show potential in increasing medical knowledge and improvement in health related quality of life
2	Being educated and being knowledgeable about epilepsy empowers parents to be an advocate for their child
3	Being educated about epilepsy made parents realise what knowledge they did not possess and caused them to seek for more information
4	Young people need accurate information about epilepsy to aid psychosocial adjustment
5	Young people need practical advice about social lifestyle management but think that healthcare professionals are only interested in medical management of epilepsy
6	Parents need practical advice but think that healthcare professionals are only interested in medical management of epilepsy
7	Young people do not receive the right information, at the right frequency and at the right time during their teenage years
8	Young women are not consistently receiving or remembering gender specific advice
9	Misinformation leads to misconceptions and uncertainty about epilepsy, and inability to cope with stigma
10	To be able to self-care and be independent of their parent, young people realise they need to know more about epilepsy to take responsibility
11	Young people do not know HOW to ask questions about their epilepsy
12	The clinical encounter mainly acts as a barrier to information exchange
13	Healthcare professionals lack facilitative skills of working in partnership with young people, with or without their parent present
14	Lack of effective partnerships and interruptions of care are having a detrimental effect on information exchange and knowledge use by young people
15	Parents are unaware of what epilepsy knowledge they do not have

### Ethical considerations

Ethical approval was obtained from the local NHS research ethics committee. Procedures adhered to standard research governance arrangements for data protection and safeguarding of vulnerable people. Young people and parents were reminded of the requirement to maintain confidentiality if participating in data collection activities with other parents or young people.

### Researcher/practitioner and reflexivity

In this Case study a researcher/practitioner (SL) conducted the interviews and focus groups and was known to some, but not all, of the participants (young people with epilepsy and their parents). Young people under the age of 16 years and those still attending the joint teenager clinic and/or waiting for an appointment in adult clinics would not have met the researcher/practitioner prior to data collection. Young people who attended clinics in adult services at the time of the study had either met the researcher/practitioner as epilepsy nurse specialist on one previous occasion, or would subsequently meet her in her role as epilepsy nurse specialist at their next appointment. Young people who knew the researcher/practitioner as their epilepsy nurse specialist appeared to be more at ease and appeared to have no apparent difficulty talking in detail about their experiences. In contrast, participants whom the researcher/practitioner had not met before initially appeared less forthcoming. The researcher/practitioner was however able to use their epilepsy-specific knowledge and communication skills to encourage participants to talk freely. Following data analysis, the researcher/practitioner had no doubts that, even though her role as nurse specialist was within researcher/practitioner, the majority of participants had no difficulty voicing their personal opinion of services, and as nurse researcher she captured their opinion verbatim. This was captured by directly asking their opinion and receiving an immediate answer, and checks were put in place to ask how participants felt about being interviewed by the researcher/practitioner. SL is also a Welsh speaker, thereby enabling participants to communicate in Welsh, which was a positive factor as some young people could not find the right words to express how they felt about certain aspects of their epilepsy while conversing in English.

All young people were made aware of the researcher/practitioner’s dual role and a clear distinction was made between the purpose of meeting in a research context and the different purpose of meeting in a clinical context. The researcher/practitioner assured all participants’ anonymity and confidentiality, and made it clear that researcher/practitioner would not mind if they wanted to tell her about less positive and positive aspects of the care they received from epilepsy healthcare professionals. Clear boundaries were discussed at the start of interviews, especially concerning safeguarding and self-disclosure of issues that could potentially require action if a participant disclosed information that raised serious concerns for their safety.

The researcher/practitioner did not disclose any aspect of their personal life or use strategies to befriend participants, and used her professional child/person centred communication skills to engage participants. Participants in focus groups and joint interviews were asked to respect each other’s contribution, and not to discuss other participant’s contributions when they returned home.

A fieldwork diary was maintained to record and reflect on fieldwork experiences. Any issues were discussed with JN to clarify what influence, if any, the issue/event/action may have on data collection and interpretation. SL used a specific reflexive framework [28] to critically analyse her role as researcher/practitioner as part of the data analysis phase (please see Additional file 1 for further information).

## Results

### Participants

**Figure 8:**
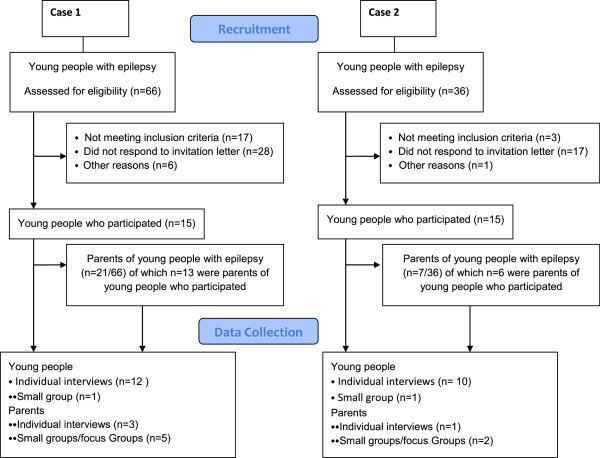
Participants Case 1 and Case 2.

Fifteen young people and 21 parents participated in Case 1, of which there were 13 child/parent pairs. Fifteen young people and 7 parents participated in Case 2, of which there were 6 child/parent pairs (Figure 8).

#### Key findings from document analysis

Implementation of national policy into practice was weak with no transition care pathway or key transition worker operating in either Case (key elements of UK national policy). 'Transition’ was not a term the majority of young people and parents in either Case recognised and they did not feel that their movement from children’s to adult services was purposeful or planned, thereby confirming problems with fidelity of transition policy when 'implemented’ in practice.

#### Themes, sub-categories, matched patterns, and what worked?

Seven themes and sixteen sub-categories (see Figure 5 for overall picture) explained young people’s and parents’ own experiences of their information needs, and communication and knowledge exchange with healthcare professionals in the various types of epilepsy clinics. We also identified many matched patterns whereby expected patterns in the theoretical framework and propositions (Figures 6 and 7 and Table 1) could be matched to observed patterns seen in the comparative embedded Case study themes. Table 2 summarises what worked, how it worked, for whom and in what context. See also Additional file 1 for additional explanation. In summary: young people in Case 2 were more willing and able to engage in a joint care model (teenager type clinic) between children and adult epilepsy services, involving a paediatrician, neurologist and epilepsy nurse meeting over months and years. Continuity and sensitive person-centred communication techniques used by healthcare professionals had a more positive impact on the engagement of young people, who were more inclined to continue with attending clinic appointments into adult services.

**Table 2:** Summary of what worked, how it worked, for whom and in what context

**What works**	**How it works (behavioural response)**	**For whom**	**In what context**
Multidisciplinary team and joint care in a single combined clinic between children and adult epilepsy services involving paediatrician, neurologist and epilepsy nurse, including:	Young people do not experience the significant change in care pathway, professionals, philosophies and frequency of clinic visits	Young people aged between 14-17 years	In Case 2
Reviewing Epilepsy and drug management early in transition	Young people and parents felt that they were having consistent coordinated expert care, and continuity of epilepsy care	Parents	Joint care between children and adult epilepsy services facilitated in children’s out-patient department of a hospital
Enabling young people to know and engage with adult healthcare professionals via children’s healthcare professionals	Young people were more actively involved in discussion about epilepsy with healthcare professionals		NHS care free at the point of delivery
Person-centred communication techniques	Young people and parents responded positively to healthcare professionals using age-appropriate facilitative skills		Specifically commissioned teenage transitional clinic from age 14-17 years (Case 2)
Continuity of care at staged intervals	Young people gained a more realistic prognosis and became more accepting of epilepsy as a long-term condition		Out-patient clinic meeting every 6 months
Continued contact with children’s services during transition	Young people felt the joint clinic in children’s services was a safe environment		
	Young people did not feel worried that healthcare professionals in adult services were going to change their anti-epileptic drugs		
	Healthcare professionals in children’s services befriending young people and making it easier for them to not be intimidated by adult healthcare professionals		
	Attending the clinic at frequent intervals with their parents if the young person wanted.		
	Increase self-confidence to self-care and manage epilepsy		
Being given age-appropriate information	Greater understanding about epilepsy as information is repeated at frequent intervals and helps overcome some but not all memory impairment	Young people aged 14-17 years	Children epilepsy clinic in Case 1 and Case 2
Healthcare professionals being responsive to the developmental age of the young person	Making links with experience of seizures (what triggers seizures)		Joint care between children and adult services Case 2
	Improves self-care by learning practical skills		
Continued checking understanding of epilepsy, lifestyle adjustment and medication management at repeated intervals	Improves self-management by improved concordance with medication	Young people aged 16-19 years	Adult epilepsy clinic Case 2
		Parents	
Healthcare professionals being receptive to young people’s varying levels of information needs throughout their teenage years	Taking responsibility increases chance of being given more independence	Young people aged 16-19 years	Routine children and adult NHS out-patient epilepsy services
		Parents	Irrespective of Case or clinic model or frequency of visits
Addressing biological, psychological, educational needs in healthcare contexts	Young people responded positively to strategies to improve retention of information – such as repeated information even if it annoyed them.	Young people aged 16-19 years	Adult epilepsy clinic Case 2 and
Early identification of memory problems and referral to neuropsychology	They experienced improved psychological coping strategies (especially at school/college)	Parents	Children’s epilepsy clinic Case 1
Early identification of psychological problems and early access to psychological services	They felt an Increase in self-confidence		
Support and advice for parents to encourage their child to safe self-care	Healthcare professionals were receptive to the individual information needs of parents and proactively responded should their child’s epilepsy change	Young people aged 14-17 years	Children’s epilepsy clinic Case 1 and Case 2
Providing accurate advice and information about epilepsy in lay language	Parents liked the emotional support as well as practical advice from healthcare professionals	Parents	Joint care between children and adult services Case 2
Providing a realistic prognosis of epilepsy as a long-term condition*	Parents responded positively to opportunities to access to parental support groups to learn from other parents		Adult epilepsy clinic Case 1 and Case 2*
	Parents self-confidence increased to enable their child to take responsibility		

* means only seen in case 2 referring to * in column 4.

Parents in Case 2 were more likely to develop the skills and confidence to encourage their children to independently and safely self-care when healthcare professionals provided individually-tailored support and advice.

Across both Cases, young people were more likely to be better educated about their epilepsy when healthcare professionals anticipated and identified their health literacy, epilepsy and lifestyle-related knowledge and information needs, and proactively facilitated targeted age-appropriate information and support. In the following section younger teenagers were aged between 14–16 years and the older teenagers were aged between 16–19 years.

### Clarity

Clarity was a theme with a matched pattern that encompassed many communication difficulties that young people and parents experienced equally in Case 1 and Case 2 during information exchange with healthcare professionals.

Young people perceived that when healthcare professionals used medical jargon they were keeping information about their epilepsy from them. This was a common matched pattern in the entire Case study data. When imparting a diagnosis of epilepsy, the issue of lack of clarity had a tendency to cause confusion, stress and anxiety. The following illustrative quote is from a small group of older teenage girls talking about when they were first diagnosed with epilepsy (Girl 1 was diagnosed in the adult clinic as a teenager (Case 1); Girl 2 was diagnosed in the children’s clinic (Case 1) as a teenager.

Girl 1: *I was told that it might not be epilepsy...............I thought I’ve just been put on tablets and no-one has discussed anything with me…*

The Doctors were just, “I don’t know, I don’t know”. They just didn’t want to say too much sort of thing.

Interviewer: *Why do you think that was?*

Girl 1: *They just didn’t want to scare me, but I got more scared because I kept thinking why; and I would have just rather know so I could have dealt with it………. because none of my family knew anything at the time so I had no-one then that I could talk to.*

Girl 2: *I had the same situation as (Girl 1). Well I just didn’t know what was going on for ages. They were just doing loads of tests and they just said epilepsy or a brain tumour or something, and when they said that it was epilepsy I threw up, but that was because it was kind of a relief as well I think, because it wasn’t a brain tumour.*

Whereas young people who received accurate information felt more at ease and were relieved to find out what was wrong with them. An older teenager who received accurate information from the time of diagnosis in the adult clinic (Case 2) after years of being considered to have psychological problems felt.

“Relieved, relieved that I knew what was wrong with me… But I’m glad it’s been diagnosed, I’m not glad I’ve got epilepsy, I mean nobody wants epilepsy, but at least I know”.

Another older teenage girl who had been diagnosed in the adult clinic (Case 2) recounted experiences voiced by other young people who felt that a clear explanation was not given.

*“I think the Consultant said it’s something to do with erratic brain waves. I didn’t really take much out of that 'cause I didn’t really know what she (doctor) was going on about”*.

She was also confused about being told that:

“You’ve got a tendency for epilepsy”… I thought basically I have or I haven’t”.

Similarly, another older teenage girl said:

“If he took the time to tell me exactly what I had, as he was telling me I have something and I felt he was not willing to tell me what that something was………… he was saying “it sounds like you have epilepsy”, but he didn’t bother going into any detail and I felt that wasn’t fair”.

The majority of young people thought that they would grow out of their epilepsy. This disconnect between perception and reality was a strong matched pattern that helped explain some behavioural consequences such as disengagement from services later in adolescence. Misinformation and/or misconceptions experienced by young people and parents was a repetitive matched pattern, irrespective of Case, especially with those whose epilepsy had begun in childhood. They had not considered and/or did not want to think about epilepsy surviving into adult life, and they believed that being on medication was for the short-term.

“They said that I could stop taking my tablet in two years, or if I wanted to stop them, but if I had a fit then I’d need to take them for another two years, I had come to terms with that”

(Younger teenage girl age, Case 1).

Being informed when attending children’s clinics in Cases 1 and 2 that they could grow out of epilepsy and then going through transition to adult services to be told that this is not going to happen was very disappointing. These particular young people and their parents appeared to be unprepared to accept epilepsy as a long-term chronic condition. One parent of a younger teenage boy who had attended the teenager clinic (Case 2) commented.

“Up until recently I was given the impression there was a good chance that he might grow out of the epilepsy, but went to the adult doctor, he is now saying 'because he has scar tissue on his brain that he won’t grow out of it’ so it’s up in the air”.

Families were upset when they realised that epilepsy was not going to go away. One father (Case 1) realised that his daughter was under the impression that she was on a course of treatment for two years and then it would be discontinued and she could get on with her life. He felt that healthcare professionals in children’s services should prepare his daughter now and not mislead her.

Irrespective of Case, the majority of parents felt that prior to their child being diagnosed with epilepsy they had no knowledge of the condition. Parents could not understand why their healthy normal child suddenly developed epilepsy. Parents spoke about the loss of their child and resented the intrusiveness of epilepsy that had completely changed their lives. Parents consistently reported that they did not know what information about epilepsy they were unaware of, and information was not readily imparted in children, teenager and adult clinics. The majority of parents did not know enough about their child’s epilepsy. The following quote shows two mothers (mother 1-older teenage daughter Case 2 and mother 2-older teenage son Case 2) talking about not receiving enough information about epilepsy from healthcare professionals:

Interviewer: *What about the epilepsy itself; do you understand what epilepsy is?*

Mother 1: *No…*.. *I have never had it explained to me, just that she has epilepsy. I don’t even know what type she has now. I didn’t realise that there were so many types. The only one I know is the one with lights.*

Mother 2: *No not really. Over the years I’ve had bits explained to me about petty mal epilepsy, and I knew what happened to him when he was having a fit, but apart from that I don’t really understand the grand mal fits. I’ve never been told anything about them….*

At the beginning I asked [questions] and then obviously they give you leaflets in the clinics that you go to. Over the years just what I’ve read and the experiences with him.

Concerned parents tried to make the most of clinic visits to improve their understanding of their child’s epilepsy, but did not know what questions to ask and medical jargon was a serious barrier to their engagement. Greater understanding was achieved when parents were able to make sense of the terminology and asked healthcare professionals to explain further. For example, a parent (Case 2) explained that for years she did not understand what healthcare professionals meant by 'identifying triggers for seizures’. When she realised 'triggers’ could be lifestyle factors, such as her daughter not having enough sleep or missing her medication, she initially felt embarrassed, and then felt angry towards healthcare professionals for not explaining it properly.

There were also gender and age-specific specific issues in both Cases with clarity of information. Most girls (aged 13-<18 years) had yet to receive information, or could not remember receiving information, about contraception and pregnancy. Girls aged 16–17 years who had not yet received reproductive health information thought that the ideal age to start discussing gender specific issues was 14 years of age.

*“I was talking to the nurse (practice nurse at GP surgery) last week about it all (contraception) and she was saying that if I wanted to have a baby I would have to go the Hospital and go for pre-something. I would have to go there (epilepsy clinic) for counselling”.* (Older teenage girl, Case 1)

The majority of girls (aged >18-19 years) had received some information at various clinic appointments in children’s and adult services but could not be specific about what they were told. Four older teenage girls were pregnant (unplanned) during their interviews (1 Girl in Case 1 and 3 Girls in Case 2) One girl (Case 1) still attended children’s services, none had attended a teenager clinic (Case 2) , and three had just started attending adult services (Case 2). None could accurately remember reproductive health information given to them pre-pregnancy. Two said that their oral contraceptive pill failed. Three out of the four girls remembered being told by the nurse not to become pregnant whilst taking their medication due to the risk of congenital malformations. One girl (Case 2) knew at the time of receiving this information in the adult clinic that she was pregnant. After her appointment, and due to fear of her medication harming her baby, she stopped taking her medication without remembering and understanding the risks associated with seizures. This was due to her usual memory impairment rather than typical forgetfulness of teenagers. She remembered that:

“Babies were born with spina bifida and stuff like that, so I was dead wary and came off it (anti-epileptic drug medication) straight away”

Her mother was unaware of the pregnancy and had not realised her daughter had suddenly stopped taking her epilepsy medication. She spoke of her own concerns in her own interview about finding out that her daughter had stopped taking her medication without telling her. All the girls worried about starving their baby of oxygen during a seizure and about harming their baby should they fall. They did not know if they could give birth 'naturally’. Following the birth they were concerned about the risk of dropping their baby because of experiencing myoclonic jerks, whether they could breast feed their baby because of their anti-epileptic drug medication, and a 'big issue’ was knowing if their baby would inherit epilepsy.

### Communication barriers

Communication barriers encompassed issues such as young people not knowing what questions to ask healthcare professionals about their epilepsy, what they needed to know about their own epilepsy, and parents being unable to seek information with their child present. Young people’s experiences were similar irrespective of the Case. A matched pattern within the theoretical framework confirmed that most young people did not know what epilepsy was and/or the name of the seizure(s) they experienced. Some young people compensated by having their parents communicate with healthcare professionals. One older teenage girl (Case 1) explained:

“When they (parents) come out with the stuff you think of it and they [healthcare professionals], when they tell you, you just understand it a bit more”

A matched pattern confirmed that some young people were afraid of receiving some epilepsy information, developed an inability to adjust, and were in fear of being alone in case something bad happened. Their own experiences of seizures were frightening, and other peoples’ responses to witnessing seizures confirmed their belief that it was frightening.

Some young people were quite specific about what issues surrounding epilepsy they were willing to discuss and were not afraid of discussing them. Some young people were afraid of receiving epilepsy information and did not want to discuss certain issues with parents and/or healthcare professionals. Young people pretended that they did not have epilepsy, wanted to *“forget about it”,* and talking about it made them worry, depressed and upset.

“*I just used to blank it out [epilepsy] until it was necessary I suppose*” (Older teenage girl, Case 2).

“*I didn’t want to speak to anyone about it at all [epilepsy] and I was just really upset all of the time*” (Older teenage girl, Case 1).

“*It [epilepsy] just made me feel depressed really, it was all I could ever think about, because everyone else around me was doing normal stuff and I felt like I was being left behind*” (Older teenage girl, Case 1).

“There’s been some points when I’ve just had too much, that I will just go to a friend and mainly cry……..just find it hard to speak to some people that don’t know about it [epilepsy], and you just don’t feel you want to bore them by going on about it”

(Older teenage girl, Case 1)

Parents found it difficult asking questions, and were frequently unable to seek information with their child present. One common concern was the risk of fatal accidents and sudden death due to seizures. Young people appeared less worried about this in comparison to parents. For example, a mother of an older teenage boy (Case 2) could not ask questions as she was afraid that the information might be frightening to both of them:

“I know we can ask (epilepsy nurse) and (epilepsy nurse) comes up with a lot of it, but there is still a lot of unanswered questions, and I do know I can’t ask them questions unless my son wants them to be asked, there’s a fear there with him”.

Some parents were too afraid to ask about the risks associated with seizures The following quote shows two mothers (mother 1- older teenage daughter Case 2 and mother 2-older teenage son, Case 2) talking about these issues:

Mother 2: *I would always say to the doctors that he doesn’t take his tablets and they would say well you do know how important it is (son), and then that’s it.*

Mother 1: *The same (daughter) just sits there and I do all the talking.*

Mother 2: *I used to find as (son) was growing up that there is some things that you don’t want to say in front of them, or anything that you don’t know or not sure about. Sometimes you don’t want to ask because the children are with you*.

Interviewer: *What do you think will happen?*

Mother 2: *I don’t know, but I think it would scare them*

Interviewer: *If (son) didn’t take his tablets and had a fit then what are the chances that he would die?*

Mother 2: *I would be frightened of saying something like that in front of (son).*

Interviewer: *Why?*

Mother 2: *I don’t want to upset him I suppose.*

### Continuity of information

Continuity of information encompassed the importance of young people remembering facts through repeating information at staged clinical intervals and the negative impact that lack of continuity of information had on the behaviours of parents and lives of young people.

Remembering epilepsy knowledge was enhanced through repeating information at staged clinical intervals, and helped some young people to become more knowledgeable about lifestyle management. A matched pattern was seen whereby young people explained that the person centred style of communication and information exchange with healthcare professionals in the joint teenager clinic in Case 2 helped them understand more about their epilepsy and motivated them to want to improve their self-management skills. This personcentred style of communication was facilitated by both children and adult healthcare professionals. Young people learned that knowing more about specific aspects about medical management actually provided practical advice which improved their self-care management. Learning practical skills to self-care also came from young people making links with their own experiences to medical, epilepsy and lifestyle information which they exchanged with healthcare professionals. For example, being informed by healthcare professionals that sleep deprivation as were missing medication, times of stress, and those with photosensitive epilepsy avoiding flickering of light such as when using a laptop and computers in college, enabled young people to better recognise their own seizure triggers and learn how to avoid them. Girls said that knowing there may be an increased risk of seizures at times of menstruation allowed then to be pre-prepared for them. Being informed about triggers which may have provoked their seizures enabled young people to make more links and reflect upon their own lifestyle in order to understand what they have to do to minimise the risk of seizures. This resulted in young people having the practical knowledge needed to self-manage their epilepsy. Most young people wanted their parents to be present to remember the verbal information. Self-care knowledge needed to be repeated during subsequent frequent clinic appointments in order to be effective as some young people were forgetful due in part to memory impairments:

*“I need something told to me three times before I take it in……..perhaps be told something and then read it, go through it, you know”* (Older teenage girl, Case 2)

Children and young people wanted verbal information given by healthcare professionals to be backed up with written materials. They were more interested in the format, presentation and relevance of information rather than the type of information, such as information in leaflets and books versus DVDs and web resources. Repeating information at times annoyed some young people, however the majority benefited from this approach of repetition of information as it strengthened recall and understanding, enabling ability to self-care. Evidence for this came from young people themselves.

Irrespective of Case, a matched pattern within the theoretical framework confirmed that lack of continuity of information from healthcare professionals resulted in some parents imposing unnecessary restrictions on their child’s life and, in particular, social activities. An informed parent was more likely to give their child more autonomy, or make more reasonable conditions to autonomy in their child’s self-care and epilepsy management. Not having accurate information led to young people and parents finding epilepsy related information via the internet. The majority reported seeing 'worst case scenarios’, and parents not having accurate information about risks and safety led to young people reporting restrictions placed on them by their parents. For example, one girl (Case 1) explained that she had to wait 3 months to receive information after being given a diagnosis of epilepsy. Her mother was extremely concerned about her safety and not knowing what to do resulted in her not allowing her daughter to be left alone or receive the appropriate information. A young person reported:

*“It (epilepsy) made me feel like I couldn’t do anything and nothing really mattered”* (Older teenage girl, Case 1)

Some parents with insufficient knowledge of epilepsy, and/or having not experienced the process of transition, feared the worst and felt they did not have the skills to encourage safe self-care. One older teenage boy (Case 1) felt annoyed to be on *'constant surveillance’*, which meant that his parents were constantly watching him and he was not allowed to go out with his friends. Like others, the following young person also felt restricted:

“I think my mum keeps me at home more…I am only allowed to go to College, and that’s only if I get a lift off my friend. She wouldn’t say you’re not going but she would be worried to death…..she texts me every hour, she won’t let me go to the shop or anywhere on my own”

(Older teenage girl, Case 1)

### Disengaging from healthcare

Disengaging with healthcare professionals and services was linked with young people and parents’ first experience with healthcare professionals in the hand-over clinic (Case 1). Once in adult services (Case 1) young people also felt forgotten by adult healthcare professionals due to infrequent clinic visits, which were fewer than when following the children’s epilepsy care pathway.

Parents felt that healthcare professionals in the handover clinic lacked facilitative skills when communicating with their children (they used medical jargon, did not involve equal participation/discussion, and appeared not to listen and respect the opinions of young people). The outcome was that some young people disengaged with healthcare professionals and subsequently the adult clinic (i.e. they refused to attend). The following quote shows a mother of an older teenage boy recounting, in Case 1, her first experience of a hand-over clinic:

Mother: *The adult team (adult hospital-teenager clinic), I mean (son) was to me a child, and it wasn’t English and they (healthcare professionals) weren’t telling us plain what it was, it was all long words and I think if it wasn’t for (epilepsy nurse) then I think I would have hit a few people.*

Interviewer: *Right, so they used jargon, did they?*

Mother: *Not what you could understand, if it’s something new and you’ve never heard about it before.*

You just want it plain, but we did learn a lot on the internet and we (mother and son) did debate the decision about going somewhere else, and who he’s with now is absolutely fantastic, because he speaks to you.

A matched pattern was seen whereby young people felt that adult services in Case 1 had forgotten about them as they were not seen as frequently as they had been in children’s services. Young people perceived that healthcare professionals did not care about them, and there was a tendency therefore for young people to disengage with healthcare services when they felt like this. Young people who had frequent interruptions to their care felt they were not seeing healthcare professionals at the right time during their teenage years. The 'right time’ was defined by these young people as times when they needed information about epilepsy related issues such as seizure occurrence, side-effects from medication and wanting to know about driving legislation.

One father in Case 1 explained that it had been over eighteen months since his son was last seen in the epilepsy clinic and felt *“a little bit out in the cold”*. His older teenage son confirmed this in his own interview, and the following quote shows that he felt adult healthcare had forgotten about him and did not care about him; he perceived this from the first contact:

Boy: *The doctor (paediatrician) I had was really good and he would sit there, and even if he had other appointments he would make sure we were finished. He wouldn’t try and get us out, and he was always really good at answering any questions that I had; he was great but this one (he made a growling noise)*

Boy: *Yeah… I’ve only seen him the once when I was transferred to him, but he’s just our opinion of him is so low because he has gone so long without contacting us. That doctor I had before, he was really good and now I’ve been handed over to this one and he seems like he is doing it because it is a job, but the other doctor cared about the patient…. He wouldn’t just tell you what you wanted to hear to get you out of the room; he would give you an honest answer………………….*

Well with him it was sort of like we walked in and he introduced himself, asked me a few questions, and then I was out and I’ve not heard from him since.

In Case 1, young people in children’s services waited until they were handed-over to adult services, and one young man had an opinion about when and how it would be most appropriate to transition to adult services. He describes his ideal service of 'an in between clinic for teenagers’, for example, he stated:

“When I was in the hospital, sitting in the children’s waiting room, I thought why are all these little kids running around and it was really annoying, but I was the oldest one there. So I think sixteen would be too early to go straight to the adult one, but I think personally it would be a good idea to have an in between one for teenagers so they wouldn’t have to sit with all the little kids, but they wouldn’t be sitting with the adults and getting intimidated either”

(Older teenage boy, Case 1 children’s services)

### On-going engagement with healthcare professionals and services

The positive impact of continuity of care and positive rapport were constant matched patterns in young people and parent’s experience of communicating with healthcare professionals. On-going and beneficial engagement was helped by befriending and continuity of care. Young people who entered the teenager clinic in Case 2 at an early age (14–15 years), and were befriended/supported by a multi-disciplinary team that included the children’s epilepsy nurse appeared more confident and able to communicate, and this confidence continued when engaging with adult services. Over time in the teenager clinic both parents and young people felt that young people developed confidence to participate in discussion surrounding their epilepsy. The majority of young people were however apprehensive about moving from the children’s to a teenager clinic. The presence of the familiar paediatrician eased the process:

*“I felt a bit shy at first moving … because I have been with that doctor for years and I was used to him, moving on was a bit scary”* (Older teenage girl, Case 2).

Young people referred to the neurologist in the teenager clinic (Case 2) as the epilepsy doctor and felt able to ask questions as they perceived that this doctor was the source of information about epilepsy:

*“We just covered pretty much everything….it was more like a discussion….kind of went both ways”* (Younger teenage girl, Case 2).

Having discussions in this way and being befriended/supported by the children’s epilepsy nurse and paediatrician in the teenager clinic in Case 2 enabled young people to have confidence to ask more about their epilepsy. Parents witnessing their child interacting with healthcare professionals had more confidence in allowing their child to take more responsibility in self-management. Young people attending the teenager clinic demonstrated that they were taking responsibility by independently making contact with healthcare professionals to discuss aspects surrounding their epilepsy and management.

Young people who saw the same healthcare professional, who was responsive to their needs, at staged frequent intervals, developed a positive rapport. The majority of young people expressed fond memories of the relationship they had developed over the years with paediatricians who managed their care. Parents mirrored their child’s comments about the consistency of seeing the same doctor, attending appointments responsive to their needs. For example, a mother of an older teenage son (Case 1) reported that healthcare professionals in children’s services had effective facilitative skills, and directly involved her son in facilitated communication and information exchange:

“He (doctor) would involve (son) with the conversation and if he didn’t understand then he would explain it to him in great depth in a way he understands”.

Even when older, many young people said that being able to bring parents into the clinic room helped. They felt that if they forgot or did not understand what had been said then at least they could turn to their parent for the information. Including parents during consultations seemed to improve family functioning and young people could see that this alleviated their parents’ stress and worry:

*“They wanted to listen to what I said (healthcare professional), how I felt, it was very good…I was able to ask questions and get answers to them, was able to bring my mum because I felt she needed to be there so she can understand exactly what needed to be known as well”* (Older teenage boy Case 2).

However, young girls felt they could not disclose unplanned pregnancy within this context. Young people feared change and wished to be involved in decision making, especially concerning drug management. Young people whose epilepsy was fairly well controlled and who transferred from children’s to adult services worried that adult services would make changes to their treatment and their epilepsy may worsen. For example, some young people still attending children’s services voiced concerns:

*“With changing doctors………the new doctor might do, like if I tell him I haven’t had fits for however long it is, 'cause I’m not having them right now, he might decide to take me off them and I will go doolally again”* (Older teenage boy, Case 1)

### Shutting down communication with parents

Shutting down communication with parents occurred when young people took ownership of their epilepsy in an overly self-protecting way and only talked about epilepsy with their parents and peers when they had to. Young people aged 16–19 years (In Case 1 and Case 2) who were given responsibility to self-care (for example, fully responsible to remember to take their medication and order repeat prescriptions) stopped talking to their parents about epilepsy unless they had to. Parents would only hear their child talk about epilepsy when attending the adult epilepsy clinic, and only if young people permitted their parent to accompany them. Some young people would use this opportunity to discuss underlying issues not spoken of at home, and/or appreciated times when healthcare professionals would bring up these issues without prompting. From the young people’s perspective, the majority did not speak to their parents about their epilepsy unless a seizure occurred. Others would only talk to their parents if they had to:

*“We don’t really talk that much about it(epilepsy); I think only if there is like an issue or a situation comes up where the epilepsy is a factor that affects it, so like going out”*(Younger teenage girl, Case 2).

Young people interpreted that if they told their parents that they felt unwell it would cause their parents to be stressed, and therefore tended to keep any epilepsy related issues to themselves. Some young people could not talk to their parents about their epilepsy:

*“They talk to me about it [epilepsy] but I don’t talk to them”* (Older teenage girl, Case 1).

### Negative impact of stigma enacted by peers

A matched pattern confirmed that stigma originating from peers had a significant impact on the behaviour of girls and boys, which may reinforce negative feelings about continued engagement with services. Inability to cope with stigma, especially for girls, originated from being stigmatised by peers at school and work. More girls than boys participated in this study so it was not therefore possible to determine whether there were any differences between Cases.

*“Someone came up to me the other day and they said do you remember that time when you had a fit and I said 'no’ 'well we call it a funky chicken’ and I said 'why?’* (she mimicked flapping her arms to demonstrate what the other person did)..........*I didn’t like going to school if I had one so I would stay at home for about a week afterwards…. Because I was scared what people would say”* (Older teenage girl, Case 1)

Across both Cases, proactive acknowledgement by healthcare professionals and addressing biological, psychological, educational needs enabled young people to feel that they were being seen as a young person and not a young person who has epilepsy. Young people were more inclined to stay engaged with epilepsy services if they could experience some tangible benefits from continued attendance.

### Post-hoc theory development

Expected patterns in the theoretical framework and propositions that matched with observed patterns are shown in red in Figures 6, 7 and Table 1. As this was a qualitative Case study, we were not able to substantiate whether age-appropriate and targeted information delivered in ways that take account of individual needs, and being knowledgeable (ie improved health literacy that takes into account any mild to moderate learning and memory impairment) improved quality of life and self-esteem as outcomes were not measured in this qualitative Case study, but overall, apart from these aspects, the Framework and propositions were supported by qualitative Case study data.

In addition, with new evidence generated from the qualitative Case study, we were able to shed more light on the complexity of health literacy in this group of young people with epilepsy and their parents. Memory impairment was seen to play a very important role in young people being able to retain information. Nonetheless, some young people appeared to make rational (to them) and yet ill-advised decisions such as stopping their medication due to misconceptions. Other factors such as inappropriate communication techniques in clinic and the imbalance of 'power’ between young people, parents and professionals all impacted on health literacy and engagement. Equally, use of medical jargon by healthcare professionals resulted in young people and parents having an unrealistic view of the future and fewer skills to self-care, as shown in red in Figures 6, 7 and Table 1. We were able to further develop the theory and propositions showing that targeted age-appropriate information, delivered consistently, with continuity, and with child-centred communication approaches enabled young people to develop skills to self-manage their epilepsy which meant they were given more freedom by their parents.

### Strengths and limitations

A significant strength of this qualitative Case study is the engagement with a large sample of young people, who are known to be highly challenging to recruit at this age and stage of their lives to research studies. There are few, if any qualitative studies of this size and richness with this particular group of young people that explore transition from children’s to adult services. Of particular significance and novelty is the high number of child/parent dyads and the different perspectives they give on the child’s epilepsy self-care and management. The qualitative Case study is data rich and strengthened by its comparative and embedded design. Another significant and novel aspect is the support of qualitative Case study data (data saturation) for almost all aspects of the theoretical framework and propositions, which can now be used to inform future practice development and research. In this respect, the novel and large scale qualitative Case study fulfils the pre-clinical theoretical phase of the Medical Research Council Framework for the development and evaluation of complex public health interventions [29]. Age-appropriate and repeated communication covering epilepsy and wider lifestyle aspects and the service model were found to be interrelated and highly important active ingredients to the continued engagement of young people with healthcare professionals and services. Nonetheless, in this type of case study we were not able to determine the relative contribution of synergistic components, or to what extent a particular professional with excellent age-appropriate communication skills could overcome some of the deficits in the service model, or the extent to which they could improve the health literacy of the young person or their parents. Our next funded feasibility and pilot study aims to explore these issues further in routine practice, culminating in a randomised controlled trial of an epilepsy-team delivered behavioural intervention to promote epilepsy medication adherence [30].

The researcher/practitioner component has both strengths and potential weaknesses. On balance, the presence of a researcher/practitioner who was known to some, but not all, young people and parents, and who was seen as a healthcare professional, appeared to be positive as a high number of young people and parents were happy to participate in the study. The transcripts indicate that they were unlikely to have been inhibited in expressing their forthright views when interviewed by a researcher/practitioner. Nonetheless, there is always a possibility that particular respondent views and experiences may only have been expressed to a person completely independent of their clinical care. The qualitative Case study was designed not to include healthcare professionals as responding actors as their perspectives are more adequately represented in current literature and the most significant gap was child and parent views and experiences.

## Discussion

The process of transition from children’s to adult services is potentially highly disruptive, with the possibility of rupturing the therapeutic relationship leading to disengagement from professional care. The design and continuity of the transition process and person-centeredness of the receiving adult service did affect the way in which young people behaved. Many young people were entering adulthood and the adult clinic lacking in skills to take responsibility for their own epilepsy self-care and management. Healthcare professionals in adult services expected young people to attend clinic on their own, and parental influence to decrease. Most young people and parents however lacked important basic epilepsy knowledge and had no clear understanding of epilepsy. Epilepsy and/or experiencing seizures had a significant impact on young people’s memory, psychological adjustment, social lives, and educational (school/college) achievement, which were not fully understood or generally taken into account by health care professionals or in the literature. Studies included in our systematic review [6] sometimes mentioned, but gave little weight to, memory impairment. When synthesising evidence across studies we were able to see the bigger picture and gave greater prominence to the importance of memory impairment in our theoretical model and propositions, which was supported by qualitative Case study data. More acknowledgement and effective strategies are required to help young people manage their memory impairment alongside their epilepsy self-care and management. Clinical implications of memory impairment are discussed in further detail below.

A multidisciplinary team and joint care in a combined clinic delivered over months and years, facilitated by children’s and adult healthcare professionals and a children’s epilepsy nurse had more, but still insufficient, active components that helped improve young people’s understanding of epilepsy and on-going engagement with adult healthcare professionals.

Facilitation of age-appropriate communication and continuity of care also enabled healthcare professionals to make better judgements about the epilepsy health literacy of young people and their parents. Young people fully or partially disengaged from services as a behavioural response triggered by healthcare professionals lacking facilitative and person centred communication skills (seen more in Case 1). This situation is dangerous as young people opted to 'go it alone’ with inadequate epilepsy knowledge and increased risk to their life and well-being.

Other studies report deteriorating health [31] and disengagement from adult epilepsy services during transition [32], but do not identify the reasons as to why young people disengage. This study fills an important gap in knowledge as young people disengaged with epilepsy services in response to both an inappropriate service model and communication approach, which were seen as interdependent active synergistic ingredients influencing their behaviour. The theoretical framework hypothesised and qualitative Case study data showed that, irrespective of the presence of memory impairment, the use of medical jargon led to young people being unknowledgeable about epilepsy and they developed misconceptions, resulting in not accepting epilepsy as a long-term condition, and lacked skills to self-manage their epilepsy. The joint teenager clinic facilitated over months and years during transition enabled young people to not experience the disruption in care pathway, professionals, philosophies and frequency of visit. Active ingredients that appeared to influence young people to engage with services included young people and parents feeling they were having consistent coordinated expert care and continuity of epilepsy care. Young people who were more actively involved in discussions about epilepsy responded more positively to healthcare professionals using age-appropriate facilitative skills. Young people gained a more realistic prognosis and became more accepting of epilepsy as a long-term condition.

Problems such as lack of epilepsy knowledge, understanding the diagnosis and social lifestyle implications appear to be little understood contemporary epilepsy self-care and management issues by young people and healthcare professionals [33]. Qualitative Case study findings stress the importance of recognising young girls not as children but as young women needing gender specific information. The perceived lack of reproductive health information and unplanned pregnancies is concerning and supported by evidence from a new UK survey whereby only 38% of girls reported being given information/advice about pregnancy [34]. A follow-up study by Wirrell et al. [35] comparing a control group of children with childhood absence epilepsy with a non-neurological chronic medical condition identified that young women with epilepsy had higher rates of unplanned pregnancy.

An epilepsy-specific factor was the importance of memory impairment and its impact on health literacy, which was not always obvious to healthcare professionals, and which remains poorly understood in the context of teaching young people to self-care and manage. Young people frequently could not remember verbal information or what they read in leaflets or on the internet. There are few, if any, interventions designed to counteract memory impairment in developing epilepsy self-care and management skills. A review reported that epilepsy educational programmes for young people improved knowledge, but there was little evidence about impact on quality of life, and behaviour change in the long-term was poor [36]. Continuity of care and repeating information at staged intervals in the current qualitative Case study helped, but new and better interventions and strategies are needed to support young people to live safely with memory impairment.

This qualitative Case study was not designed to measure if knowledge of epilepsy improved young people’s quality of life and self-esteem. An observational study is currently in progress to fill this gap [37]. There is also a debate in the wider literature as to whether self-esteem is the best concept to measure. Although self-compassion did not feature in the epilepsy-related literature when developing the theoretical model and propositions, some researchers have put self-compassion forward as a more appropriate indicator of a healthy attitude and healthy lifestyle in relation to self-management [38,39]. We will explore self-compassion in future studies.

Some young people were striving for independence but were held back by their parents because of their epilepsy. Qualitative Case study data provides new and interesting explanations for further investigation. Similar to less rich evidence from audits, the majority of parents lacked epilepsy knowledge, and found it very difficult to let go as their child entered adulthood [40-42]. The reasons as to why parents explicitly or implicitly discouraged or prevented independent self-care and management by their teenagers are little understood in the literature. We found that the problem was poorly conceptualised and understood by many healthcare professionals, who offered few interventions other than increased information and support to enable parents to let go. Young people were concealing certain aspects about their condition, and their self-care and management was not always concordant with epilepsy care plans, which resulted in constant confrontation affecting parent–child relationships [43]. Young adults (irrespective of whether they have a long-term condition) generally perceive risk differently to adults, for example: smoking/drugs/alcohol, wearing helmets, driving etc. [44]. The process of differentiating young people from parents is important, especially when, as professionals, we have experience of adult patients with epilepsy in their 40s and 50 s attending clinic with their aging parents. Integrating theory into clinical reasoning may help support better understanding of these challenging behavioural issues. For example, Erikson’s [45] theory of psycho-social development on 'Identity vs. Role Confusion’ may be useful in understanding the impact of parental behaviour. Young people’s experiences from previous developmental stages are thought to stay with them and impact on experiences at future developmental stages. During normal adolescence parental roles change from being protective to supporting their child to become independent. Not promoting independence can induce a crisis with young people needing to find out who they are and what their place in society is [45]. Child and parent participation in self-care interventions may be a way of developing family-based knowledge and skills and mutual understanding, but further research is needed [46].

In an ideal world, behavioural change issues in long-term conditions management should be proactively managed by the integration of clinical psychologists and social workers into multi-disciplinary epilepsy teams. In the current global resource constrained environment, health providers are however highly unlikely to invest further in epilepsy services unless cost-neutral savings can be made elsewhere. In our experience, clinical psychologists and social workers are an invaluable but scarce resource with long waiting lists, so only those young people and parents with the most challenging problems are referred, and often too late. Our current strategy is to work with a psychologist in a research context to explore the feasibility of teaching epilepsy professionals mindfulness-based approaches for use in routine clinical practice [30] so that they can identify behavioural problems as they appear and deliver 'real-time’ interventions. With this very early mindfulness-based intervention delivered in routine practice, we hope to improve the quality of care that young people receive and reduce the need for referral to the ever stretched clinical psychology or social worker service.

Finally, as a marker of transferability, some findings, such as the need for continuity of care, are common experiences of transition expressed by young people with other long-term conditions [2,47,48]. Nonetheless, the major new contribution that this qualitative Case study makes is the rich description of young people and their parent’s experiences of different transition models, the different types of continuity they offer, and their impact on continued engagement with services. In this respect the current qualitative Case study is unique. Given the size of the study, the saturation of themes and pattern matching across cases, evidence is likely to be transferable to other similar contexts with similar age groups of young people with epilepsy, and their parents. Findings are also likely to resonate with young people of similar age groups with other long term conditions who experience similar transition service models.

## Conclusion

The transition process from children’s to adult epilepsy services is a highly complex and multifaceted longitudinal intervention with various models, and little evidence of the effectiveness of specific models. The model of transition between children and adult services does however impact in a variety of different ways on children’s continued engagement with epilepsy services and outcomes of care. There are specific active ingredients that appear to work, irrespective of the model of transition (integration of neuropsychologist to identify memory impairments and child psychologist in epilepsy team), which need to be incorporated into the most promising model. The transition model that looked the most promising was a jointly facilitated child/adult epilepsy clinic with a staged transition over many months/years with specific focus on the real problems and social realities of living with epilepsy from the perspective of the young person. Importantly, this model still respected parents as partners in their child’s care, if that is what young people wanted. Within this context the active ingredients were continuity of longitudinal care; continuity of child-centred communication and information; continuity of relationships, and preparing parents to let go. Young people disengaged with services in response to both an inappropriate service model and inappropriate communication, which are interdependent active synergistic ingredients that influenced their behaviour. Although generic active ingredients will apply to other groups; addressing memory impairment is especially important to young people with epilepsy.

Findings from this technically complex study offer new and novel evidence, and a mostly supported theory on the active ingredients to incorporate into the design of future interventions. Future research is needed to evaluate the effectiveness of complex service models and interventions to improve transition from children’s to adult epilepsy services. The findings of future randomised and non-randomised trials of behavioural interventions to promote self-care and management offer further opportunities to develop and refine the theory with evidence from validated outcome measures.

## Competing interests

SL has received funding from Epilepsy drug companies to attend epilepsy conferences. JN declare no competing interests.

## Authors’ contributions

SL and JN designed the study. SL collected data; SL and JN analysed data, developed the theory and drafted the manuscript. Both authors read and approved the final manuscript.

## Authors’ information

Dr Sheila Lewis (PhD) is a Nurse Clinician in Epilepsy working in NHS Hospitals in England and Wales. She is following a clinical academic career pathway and has recently been awarded the NISCHR Academic Health Science Collaboration (AHSC) Clinical Research Fellowship to conduct a research project: CHildren and Young people Managing Epilepsy (CHYME). The aim of her research project is to develop the evidence-base for using behaviour interventions in routine clinical practice by various healthcare professionals to improve medicine self-management at home for children and young people with epilepsy.

Professor Jane Noyes is Noreen Edwards Chair in Health Services Research and Child health, specialising in children’s health and social services research and cost consequences. She is also interested in qualitative and mixed method systematic review methodology, and is Lead Convenor of the Cochrane Qualitative and Implementation Methods Group.

## Pre-publication history

The pre-publication history for this paper can be accessed here:

http://www.biomedcentral.com/1471-2431/13/169/prepub

## Supplementary Material

Additional file 1 Researcher/practitioner and reflexivity. Click here for file
