# Supplementary material for: Effective process or dangerous precipice: qualitative comparative embedded case study with young people with epilepsy and their parents during transition from children’s to adult services
Source: BMC Pediatr. 2013 Oct 16;13:169. doi: 10.1186/1471-2431-13-169 (PMC4016204; doi:10.1186/1471-2431-13-169)
Supplement: Additional file 1 — Researcher/practitioner and reflexivity. [file 1471-2431-13-169-S1.docx]

### Further information about what worked, how it worked, for whom and in what context

### Joint care between children and adult epilepsy services involving a paediatrician, neurologist and epilepsy nurse worked for young people with epilepsy

It was evident that joint care between children and adult epilepsy services involving paediatrician, neurologist and an epilepsy nurse in Case 2 (Figure 6) was most effective for young people and parents. The observed patterns that consistently matched the expected patterns confirmed more positive engagement with healthcare professionals and services in this context. Young people who attended the joint clinic at staged intervals learnt to engage with healthcare professionals and became involved in discussion about their epilepsy. They engaged more by being befriended and becoming familiar with the healthcare professionals involved. Findings from document analysis showed that a named paediatrician in Case 1 and Case 2 took responsibility to identify young people at a younger age and shared their care until they were at the appropriate age to be transferred to the adult epilepsy clinic.

Young people said or implied that healthcare professionals within this context were using age-appropriate facilitative skills when communicating with them. Young people in turn felt befriended by healthcare professionals which increased their self-confidence. Young people and parents considered the children’s department to be a safe environment, as they were familiar with their surroundings and had age-appropriate resources in place which helped young people engage with healthcare professionals. Young people who had attended frequent clinic visits, and had opportunities to experience communicating with existing and new healthcare professionals who conveyed consistent information and treatment plans, appeared to be positive factors influencing young people to continue engaging in a more meaningful way as intended.

###

### Actively facilitated age-appropriate epilepsy-related information worked for young people with epilepsy.

Actively facilitated age-appropriate information that was integrated into routine care was found to be effective for the majority of young people. Pattern matching confirmed that the supported the majority of the theoretical propositions. Young people developed greater understanding about their epilepsy when given accurate information they could understand and was not concealed in medical jargon. Healthcare professionals who used medical jargon created an opportunity for a specific behavioural response (mechanism) whereby young people became concerned about whether they had epilepsy or not and they were also afraid that healthcare professionals were concealing important information from them.

When healthcare professionals were giving practical advice about seizure management in a language young people understood; young people were able to respond by making links with their experience of seizures and appeared to understand more about ‘triggers’ for their seizures. Being knowledgeable through understanding more about their own epilepsy appeared to improve self-management skills and concordance with medication. Document analysis confirmed that young people received resources such as information leaflets which were age-appropriate. Parents also reported that they found information leaflets for their children easier to understand.

When healthcare professionals are receptive to young people’s varying levels of information needs throughout their teenage years and they take time to check understanding of the information imparted in person centred ways, there is a better more effective relationship based on partnership to underpin self-management of epilepsy between young people, parents and healthcare professionals.

### Proactive acknowledgement by healthcare professionals and addressing biological, psychological, educational needs in healthcare contexts worked for young people

Healthcare professionals who addressed the medical (epilepsy) information needs of young people alone were not effective in their management of young people. During matching the expected patterns with the observed patterns young people needed their biological (memory), psychological and educational (school/college) needs to be addressed in healthcare contexts equally. Themes such as ‘clarity’; ‘communication barriers’ and ‘continuity of information’ matched propositions where young people perceived the clinical encounter as a barrier to information exchange and they thought that healthcare professionals were only interested in their epilepsy.

The majority of young people self-reported that since they were diagnosed with epilepsy they were more forgetful; experienced short-term memory loss; difficulty remembering information and felt that they were cognitively slower than how they were before. These biological (memory problems) impacted on their educational (school/college/work) needs. Young people developed psychological problems due to experiencing stigma enacted by peers in school/work. Young people self-reported that they had difficulty remembering and learning information that teachers imparted during lessons. Having subtle seizures and side-effects from their medication also affected what young people remembered. Due to their epilepsy and/or having seizures some young people reported that they either stayed at home following a seizure or did not go to school or deliberately did not go to school as they felt that because of having epilepsy their teachers were not interested in them.

When healthcare professionals identified early that young people had memory problems they provided strategies to improve retention of information (repeating information at staged intervals). Young people who had psychological problems and early access to psychological services had improved coping skills and self-confidence. Young people had improved coping skills when school (teachers) understood about their epilepsy due to improvement in communication between healthcare service and education. Within this context, epilepsy, biological, psychological and educational needs of young people with epilepsy are best met by a multi-disciplinary team working effectively together in clinic settings.

### Healthcare professionals providing individually-tailored support and advice for parents to encourage their child to safe self-care worked for parents in Case 2

During matching the expected patterns with the observed patterns we confirmed that parents do need continuous support and advice throughout their child’s teenage years to encourage their child to safe self-care. The themes ‘clarity’ and ‘continuity of information’ matched the theoretical proposition that parents being educated and knowledgeable about epilepsy are empowered to be an advocate for their child. Parents were unaware of what epilepsy knowledge they do not have.

Healthcare professionals who were receptive to the information needs of parents when their child’s epilepsy changed (for example, worsening of seizures despite their child taking their medication) appeared to improve parents coping ability. Parents need practical advice about epilepsy and are equally affected by medical jargon that impedes on their understanding about epilepsy. Being given an accurate prognosis about the likelihood that their child will have epilepsy long-term enabled parents to help prepare their child future needs. Parents who could see that their child understand more about their own epilepsy and are taking responsibility for taking their medication are given more independence. The authors interpreted those themes such as ‘communication barriers’ matched the proposition where lack of effective partnership and interruptions of care continuous to have a detrimental effect on information exchange and knowledge use by young people and parents. ‘Continuity of information’ between healthcare professionals and parents improved partnership and on-going care in healthcare contexts.
